# Real-world effectiveness of cytology and HPV-based screening strategy in cervical cancer screening: A cross-sectional population-based study in Chengdu, China

**DOI:** 10.1371/journal.pone.0299651

**Published:** 2024-02-29

**Authors:** Boshuang Yao, Jieru Peng, Wei Song, Liu Yang, Meng Zhang, Xia Wu, Shiyi Wu, Xiaoyu Wang, Chunrong Li, Chunxia Yang

**Affiliations:** 1 Department of Epidemiology and Biostatistics, West China School of Public Health and West China Fourth Hospital, Sichuan University, Chengdu, Sichuan Province, China; 2 Non-communicable Diseases Research Center, West China-PUMC C.C. Chen Institute of Health, Sichuan University, Chengdu, Sichuan Province, China; 3 Chengdu Municipal Health Commission, Chengdu, Sichuan Province, China; 4 Chengdu Women’s and Children’s Central Hospital, School of Medicine, University of Electronic Science and Technology of China, Chengdu, Sichuan Province, China; Teikyo University, School of Medicine, JAPAN

## Abstract

Cervical cancer poses a significant health challenge in developing countries, emphasizing the need for appropriate screening strategies to accelerate the elimination of this disease. This study summarized the results of a large-scale community-based cervical cancer screening program conducted in Chengdu, China, to understand the prevalence of HPV infection and cervical lesions in the population, and to compare the real-world effectiveness of two different screening methods implemented in the program. From January 2021 to December 2022, a total of 363,376 women aged 35–64 years in Chengdu received free screenings. Among these participants, 70.1% received cytology screening and 29.9% received HPV testing combined with 16/18 genotyping and cytology triage. Ultimately, 824 cases of high-grade lesions and cervical cancer were detected, with a total detection rate of cervical cancer and precancerous lesions of 226.8 per 100,000. The follow-up rate of patients with high-grade lesions and above was 98.9%, and the treatment rate was 86.6%. The overall high-risk HPV infection rate was 11.7%, with the HPV 16/18 infection rate of 1.4%. The rate of abnormal cytology results was 2.8%. The attendance rates for colposcopy and histopathology were 71.6% and 86.1%, respectively. By calculating the age-standardized rates to eliminate the different age composition between the two group, the HPV-based screening strategy had a higher rate of primary screening abnormalities (3.4% vs. 2.8%, *P*<0.001), higher attendance rates of colposcopy (76.5% vs. 68.9%, *P*<0.001) and histopathological diagnosis (94.1% vs. 78.0%, *P*<0.001), higher percentage of abnormal colposcopy results (76.0% vs. 44.0%, *P*<0.001), and higher detection rate of cervical precancerous lesions and cancer (393.1 per 100,000 vs. 156.4 per 100,000, *P*<0.001) compared to cytology screening. Our study indicates that the combination of HPV testing with 16/18 genotyping and cytology triage has demonstrated superior performance in cervical cancer screening compared to cytology alone in large-scale population.

## Introduction

Cervical cancer is the fourth most common malignancy in women and remains the leading cause of cancer morbidity and mortality among women in numerous countries globally [1]. In 2020, there were approximately 604,000 new cases of cervical cancer and 342,000 deaths worldwide, with the age-standardized incidence of 13.3 per 100,000 and mortality rate of 7.2 per 100,000 [2]. Notably, more than 80% of new cases and deaths from cervical cancer occur in low- and middle-income countries or regions, resulting in a significant impact on their health and hindering local social and economic development [3]. The age-standardized incidence and mortality rates of cervical cancer in China were lower than the world average, with rates of 10.7 and 5.3 per 100,000 respectively, however, China still accounted for approximately one fifth of all new cases (109,700) and deaths (590,000) worldwide in 2020 due to its large population base [2]. Since the 21st century, there have been increasing trends in the incidence and mortality rates of cervical cancer among Chinese women, this growing burden poses a significant public health challenge that requires attention and resolution [4].

Regular screening can identify cervical precancerous lesions, offering opportunities for treatment and effectively reducing the incidence and mortality of cervical cancer. Cytology has been the most commonly used screening method in recent decades for its relatively high specificity, simplicity and affordability [5]. As of 2020, 48 of the 139 countries or territories with cervical cancer screening recommendations have proposed HPV-based screening as the primary screening method, because HPV testing is more sensitive in detecting high-grade precancerous lesions than cytology [6]. Moreover, HPV testing is not affected by the quality of the slide, the experience of the cytologists, or other subjective factors [7]. However, studies have shown that HPV testing alone has a relatively high false positive rate, which can lead to over-diagnosis and over-treatment. As a result, HPV testing is often combined with cytology to improve the screening effectiveness [8].

China’s cervical cancer screening program has witnessed a transition in screening methods from acetic-acid/iodine staining to cytology and the latest HPV testing since its launch in 2009. Additionally, the target population has expanded from rural women aged 35–64 to all women aged 35–64 living in urban or rural areas, and the screening coverage has steadily increased. However, given China’s large population and limited health resources, the current state of screening in China falls short of being thoroughly satisfactory. Until 2019, the coverage rate for screening among women aged 35–44 in China was 43.4%, 39.6% for women aged 30–49, and 36.8% for women aged 35–64, with even lower coverage in rural and central and western China, falling well below the WHO’s target of 70% [9, 10]. It is imperative to find suitable screening strategies to improve coverage in China.

In 2019, China introduced the Healthy China Action Plan (2019–2030), which aimed to achieve an 80% cervical cancer screening coverage by 2030. In response to this initiative, Chengdu, a city in Sichuan Province in western China, launched its Healthy City Project in 2021, which includes free HPV-based cervical cancer screening for female residents aged 35–64 years, whereas Chengdu previously used a primarily cytology-based screening strategy. As there are limited studies on the real-world effectiveness of HPV-based screening strategies in developing countries with large populations, we conducted this study to summarize the results of this large-scale community-based cervical cancer screening program conducted in Chengdu, to understand the prevalence of HPV infection and cervical lesions in the population and to compare the real-world effectiveness of two different screening methods.

## Methods

### Data source and ethics approval

This study retrospectively analyzed the results of cervical cancer screening in women aged 35–64 years from January 1, 2021 to December 31, 2022 in Chengdu, and the data was extracted from archived records of the Cervical Cancer Screening Registration Database from the local health administrative department. The data for research purposes were accessed on August 15, 2023, with the approval of the local health administrative department. This study was approved by the Ethics Committee of West China Fourth Hospital and West China School of Public Health, Sichuan University (No. Gwll2023141). All participants data we accessed and analyzed were completely anonymous and did not contain information that could identify individual participants, thus the requirement for informed consent was waived by the ethics committee.

### Participants

The study included women who attended cervical cancer screening in Chengdu between January 2021 and December 2022. Female residents aged 35–64 years could obtain free gynecological examinations and cervical exams at designated hospitals or primary maternal and child health care hospitals. Inclusion criteria for participants were women aged 35–64 years with sexual history. Exclusion criteria included: 1) women with a history of total or subtotal hysterectomy or cervical surgery, 2) pregnant or menstruating women, and 3) women who had used vaginal medication or engaged in sexual activity within 72 hours prior to screening.

### Screening strategies

Between 2021 and 2022, designated hospitals and primary maternal and child health care hospitals in Chengdu that were responsible for free cervical cancer screening gradually transitioned from cytology to HPV-based screening strategy. The screening strategies used by participants largely depended on whether the screening organizations visited had completed the transition of the primary screening methods, with those participating in the early phase of the program mainly using cytology and, from the transition period onward, an increasing number of people adopting the HPV-based screening strategy.

Women participating in the screening first underwent routine gynecologic examinations, followed by collection of cervical specimens by gynecologists. Collected specimens were stored as needed and sent to designated laboratories for cytology or HPV testing within a specified timeframe. Cytology adopted the liquid-based method, and HPV testing was based on the PCR method using kits that required the ability to detect at least 13 high-risk genotypes (16, 18, 31, 33, 35, 39, 45, 51, 52, 56, 58, 59, 68) and the ability to report positive results for types 16/18 separately. All procedures for specimen collection, storage and laboratory testing were performed according to the kit instructions.

The two different screening strategies were implemented as follows: 1) Participants underwent cytology screening alone, those with abnormal cytology results were then referred for colposcopy. 2) Participants who tested positive for HPV 16/18 types were directly referred to colposcopy, while people infected with non-16/18 HR-HPV were triaged with cytology, further, those with abnormal cytology triage results were referred to colposcopy.

Finally, participants with abnormal colposcopy findings were required to undergo histopathological diagnosis. If diagnosed with high-grade squamous intraepithelial lesions (HSIL) or worse through histopathology, they were promptly notified to receive treatment.

### Explanation of the screening results

Cytology results were reported based on the Bethesda System (TBS), atypical squamous cells of undetermined significance (ASC-US) or worse results were identified as abnormal or positive cytology results [11]. For the HPV-based screening strategy, HPV 16/18-positive, non-16/18 HR-HPV-positive combined with cytology triage results ≥ ASC-US were defined as positive for primary screening.

Colposcopy results were reported as negative, low-grade lesions, high-grade lesions and suspicious for cancer. Low-grade lesion or worse were reported as abnormal results.

Histopathological results were classified as negative or inflammation, low-grade squamous intraepithelial lesion (LSIL)/cervical intraepithelial neoplasia grade 1 (CIN1), HSIL/CIN2-3, adenocarcinoma in *situ* (AIS), micro-invasive cervical cancer and invasive cervical cancer. HSIL/CIN2-3 and higher grades of cervical lesions were the main outcomes of interest.

### Definition of outcome indicators

The following indicators were analyzed to evaluate the effectiveness of screening:

The overall abnormality rate for primary screening = (cases with cytology results ≥ ASC-US + HPV 16/18-positive cases + cases of non-16/18 HR-HPV positive combined with cytology triage results ≥ ASC-US) / number of total participants in screening * 100%.

The abnormality rate of primary screening for cytology strategy = cases with cytology results ≥ ASC-US / number of participants receiving cytology screening * 100%.

The abnormality rate of primary screening for HPV-based strategy = (HPV 16/18-positive cases + cases of non-16/18 HR-HPV positive combined with cytology triage results ≥ ASC-US) / number of participants receiving HPV-based screening * 100%.

The HPV infection rate = number of HPV positive cases/number of people tested for HPV * 100%.

The attendance rate of colposcopy = number of cases with abnormal primary screening results who actually received colposcopy / number of persons with abnormal primary screening results who needed to undergo colposcopy*100%.

The attendance rate of histopathology = number of persons who actually received histopathological diagnosis / number of persons with abnormal colposcopy findings who needed to undergo histopathological diagnosis *100%.

The colposcopy abnormality rate = number of people with abnormal colposcopy findings / number of people who underwent colposcopy * 100%.

The detection rate of cervical precancerous lesions and cancer = number of cases with histopathologic findings ≥ HSIL/CIN2-3 / number of people participating in screening*100,000.

The early diagnosis rate = number of cases with histopathologic findings of HSIL/CIN2-3, AIS and micro-invasive cancer / total number of people with histopathologic findings ≥ HSIL/CIN2-3*100%.

The follow-up rate for treatment = number of cases actually followed up / number of cases requiring follow-up with histopathologic findings ≥ HSIL/CIN2-3 * 100%.

The treatment rate for cervical lesions = number of people treated / number of people actually followed up to treatment *100%.

### Statistical analysis

The age distribution of the screening population was described using the median and interquartile range (IQR), and the Mann-Whitney test was used to compare the differences in age distribution between the two different screening strategy groups. The screening population was divided into six groups according to age intervals (35~, 40~, 45~, 50~, 55~, 60–64), and the age composition of the two groups was further compared using the chi-squared test.

Indicators used to assess the effectiveness of screening were count variables, described as frequencies and percentages. We calculated age-specific rates to learn about the distribution of HPV infection, abnormal cytology findings, and cervical lesions across age groups. To compare the effects of the different screening strategies, we used age-standardized rates to make comparisons between the two groups, using the age structure of the total screening population (HPV-based screening population + cytology screening population) as the standard composition to eliminate the effect of different age compositions. All comparisons of rates between groups were analyzed using the chi-squared test or Fisher’s exact test. Statistical analyses were performed using SPSS 26.0, and all statistical tests conducted were two sided with a statistically significance level of *P* < 0.05.

### Potential bias and quality control

Due to the large scale of the screening program, several designated hospitals and laboratories performed the screening and testing, which may have led to some measurement and reporting bias in the results from different institutions. To ensure the quality of the screening, cytology and HPV test kits used in the screening were uniformly purchased by government departments, health care workers involved in the screening program received regular training and strictly followed the screening procedures, and designated laboratories conducted regular quality control and evaluation. Additionally, a team of specialists was established to supervise and control the quality of clinical examinations and the accuracy of information stored in the database. However, it should be noted that HPV-based screening was introduced later than cytology screening, which may have led to some unavoidable measurement and reporting bias between the two periods.

## Results

### Participant characteristics

**Fig 1:**
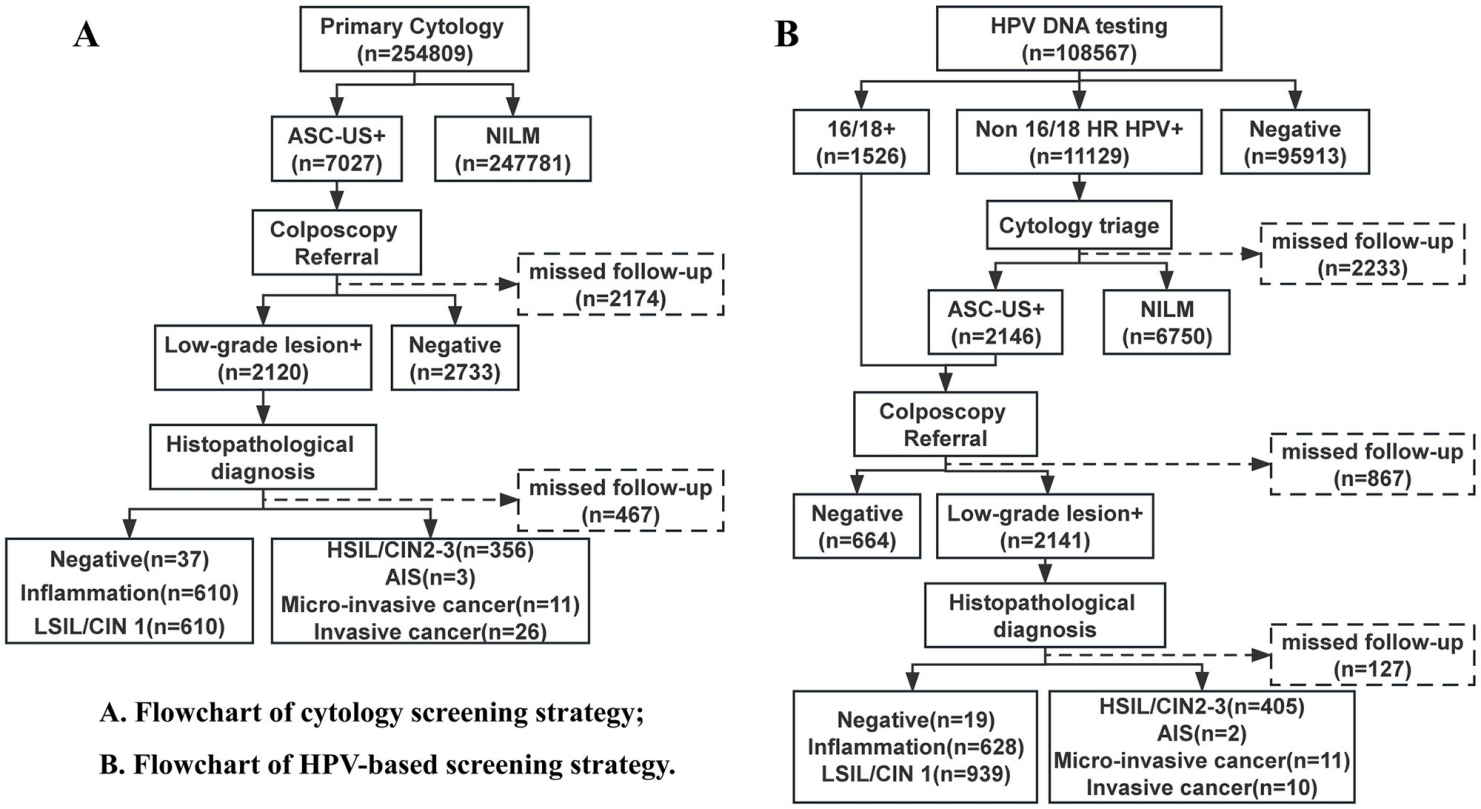
The screening strategies and workflow.

This study included a total of 363,376 women aged 35–64 years who participated in cervical cancer screening in Chengdu from January 2021 to December 2022. Among the participants, 254,808 (70.1%) women underwent primary cytology screening, while 108,568 (29.9%) women underwent HPV-based testing. Fig 1 provides additional information about participants and the two different screening procedures.

The median age of the screened population was 50 years old (IQR:43–54), and the age distribution of the participants is shown in Table 1. The population undergoing HPV-based screening was found to be younger compared to the population receiving cytology (*P*<0.001). This was mainly because of a relative higher proportion of women younger than 45 years in the HPV-based screening population. Regardless of the screening method, the highest proportion of participants was in the age group of 50–54, while the lowest proportion was observed among women aged 60–64 years.

**Table 1:** Age characteristics of the screened population and comparison between different screening groups.

	HPV-based testing	Primary cytology	Total population
Median age, IQR* **(years)**	48 (41–54)	50 (44–54)	50 (43–54)
***P* value** ^#^	***P*<0.001**		--
**Age group (n, %)**			
35–39	18,727 (17.2)	29,334 (11.5)	48,061 (13.2)
40–44	17,779 (16.4)	35,234 (13.8)	53,013 (14.6)
45–49	23,009 (21.2)	56,504 (22.2)	79,513 (21.9)
50–54	26,452 (24.4)	71,113 (27.9)	97,565 (26.8)
55–59	17,783 (16.4)	51,207 (20.1)	68,990 (19.0)
60–64	4,818 (4.4)	11,416 (4.5)	16,234 (4.5)
Total	108,568 (100.0)	254,808 (100.0)	363,376 (100.0)
***P* value** ^#^	***P*<0.001**		--

*IQR, interquartile range;

^**#**^*P* value, significance level for comparing the age distributions of the two groups using different screening strategies.

### Overall screening findings

A total of 824 patients with cervical cancer and precancerous lesions were detected among 363,376 participants, including 761 cases of HSIL/CIN2-3, 5 cases of AIS, 22 cases of micro-invasive cancer and 36 cases of invasive cervical cancer. The detection rate for cervical precancerous lesions and cancer was 226.8 per 100,000, with an early diagnosis rate of 95.6%.

**Fig 2:**
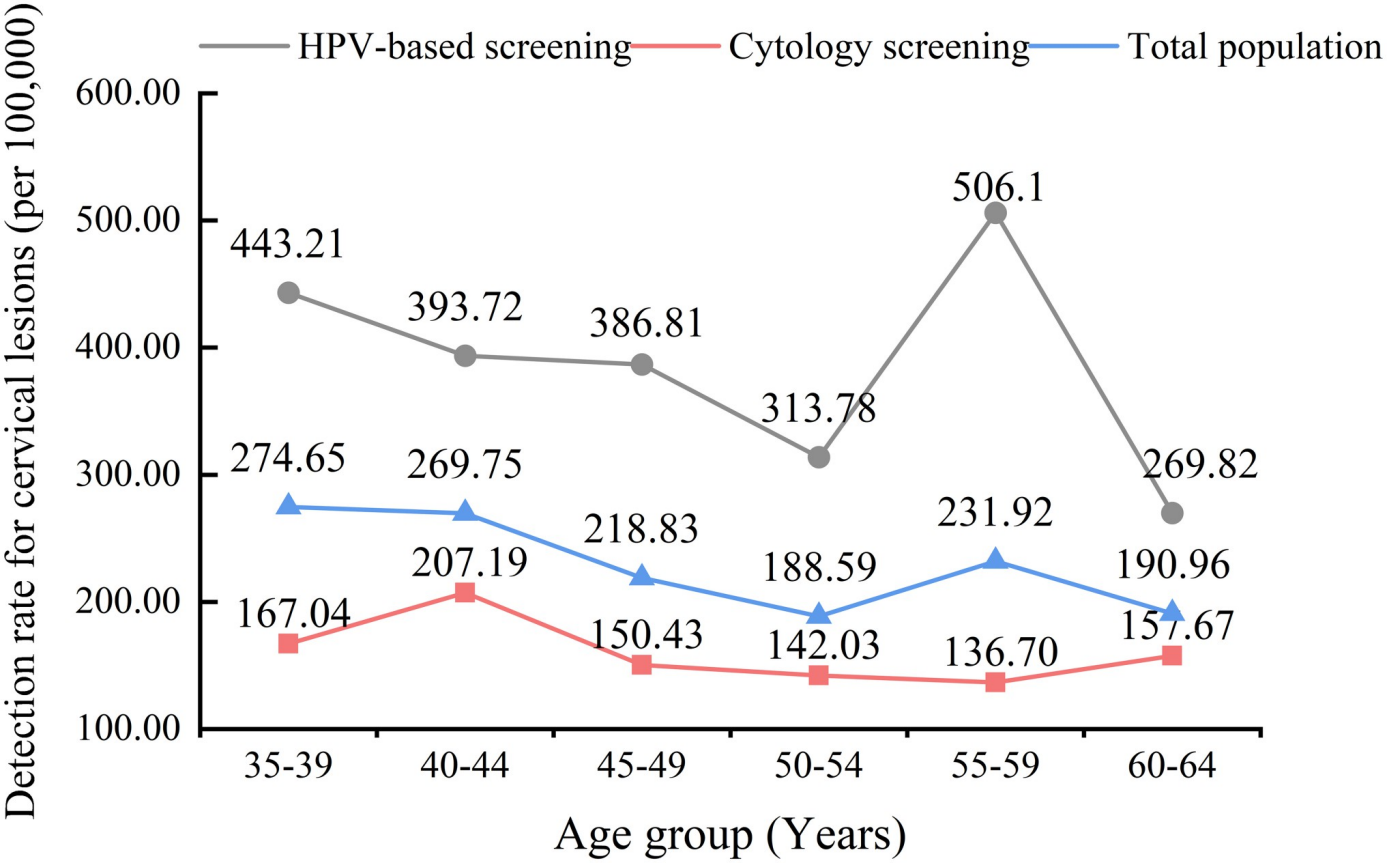
Age-specific detection rates of cervical lesions for different screening methods.

Age-specific detection rates were analyzed to understand the prevalence of cervical lesions in different age groups. It was found that the detection rates of cervical lesions varied by age in the total screening population (*P* = 0.005) and in the HPV screening strategy group (*P* = 0.022), and the difference in age-specific detection rates was not statistically significant in the cytology screening strategy group (*P* = 0.135). Fig 2 shows the trend of age-specific cervical lesion detection rates in the different groups.

### Prevalence of HPV infection and cytology screening results

The overall HR-HPV infection rate in the population undergoing HPV testing was 11.7%. Specifically, the rate of HPV 16/18-positive was 1.4%, while the rate of non-16/18 HR-HPV infection was 10.3%. Among participants positive for non-16/18 HR-HPV, 79.9% received cytology triage, and the positive rate for cytology triage findings was 24.1%. The HPV-based screening methods identified a total of 3672 cases infected with HPV 16/18 or infected with non-16/18 HR-HPV with abnormal cytology triage findings, with a primary screening abnormality rate of 3.4%. Among individuals who underwent cytology screening, the abnormality rate of cytology results was 2.8%. Overall, the prevalence of primary screening abnormalities in the total population was 2.9%.

**Fig 3:**
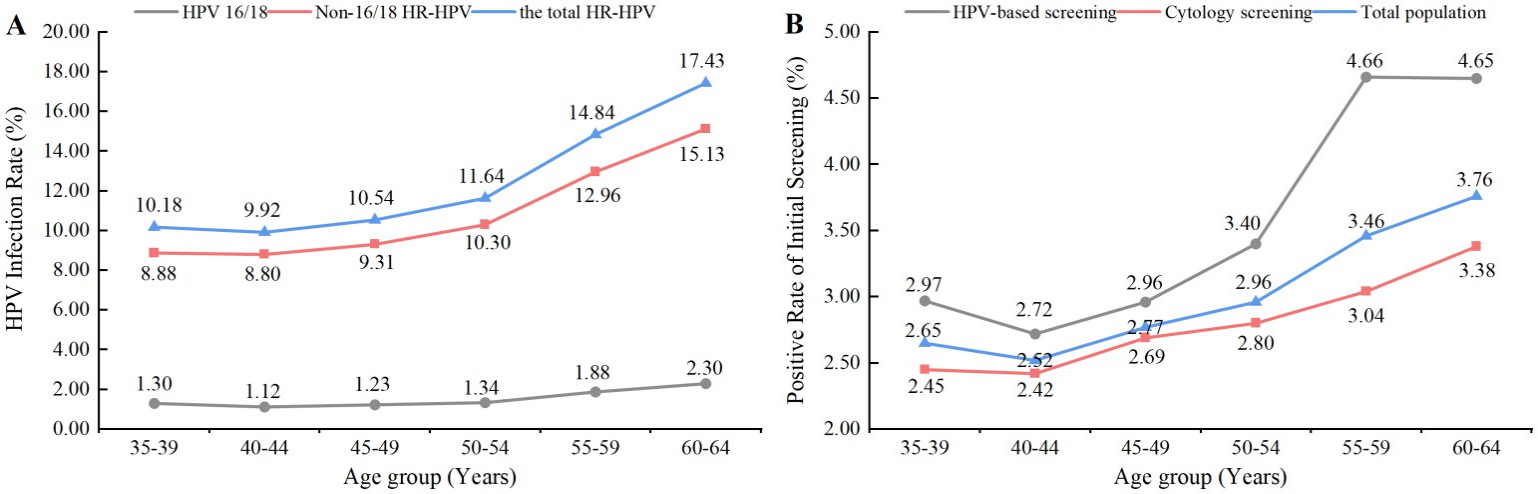
The age-specific prevalence of the HPV infection (A) and abnormal results of primary screening (B).

The age-specific primary screening abnormality rates for the two screening methods and the HPV infection rates of different genotypes in the population exhibited overall increasing trends with age (*P*<0.001). Fig 3 illustrates the trends in primary screening abnormality rates and HPV infection rates among different age groups.

### Results of colposcopy referral and histopathological diagnosis

A total of 10,699 women with abnormal primary screening results should been referred for colposcopy, however, only 7,658 women underwent colposcopy, resulting in an attendance rate of 71.6%. Among those who underwent colposcopy, the abnormal rate for low-grade lesions or worse was 55.6%. Finally, 86.1% of women with abnormal or suspicious colposcopy results underwent histopathologic diagnosis, and 824 patients with HSIL/CIN 2–3 or worse lesions were detected.

We further analyzed the age-specific attendance rates of colposcopy and histopathology, and the results showed differences by age in the attendance rates of populations within the same screening strategy (*P*<0.001), except for histopathology in the cytology screening group (*P* = 0.55). The abnormality rate of colposcopy findings also differed by age in the population within the same screening strategy (*P*<0.001). These results are summarized in Table 2.

**Table 2:** Age-specific rates of colposcopy and histopathology attendance and colposcopy abnormality findings.

Age group	attendance rate of colposcopy (%)		abnormality rates of colposcopy findings (%)		attendance rate of histopathology (%)	
	Cytology screening	HPV-based screening	Cytology screening	HPV-based screening	Cytology screening	HPV-based screening
35–39	60.7	78.5	54.8	80.8	81.2	95.5
40–44	69.1	73.9	53.8	81.8	80.8	93.8
45–49	68.5	80.9	45.5	78.9	75.7	96.1
50–54	71.7	76.2	40.3	70.8	79.0	94.0
55–59	70.7	74.2	36.8	73.2	73.6	92.9
60–64	66.3	71.9	42.2	78.9	83.3	88.2
Total rate*	69.1	76.4	43.7	76.3	78.0	94.1

*Total rate, the total crude rate for the population in the different screening strategy groups.

### Follow-up and treatment

For 824 patients with histopathologic findings ≥ HSIL/CIN 2–3, the follow-up rate reached 98.9%. Among patients who were followed up, the treatment rate for precancerous lesions and cancer was 86.6%.

### Comparison of the effectiveness of different screening methods

To assess the real-world effectiveness of the two different screening strategies, we analyzed age-standardized rates of cervical lesion detection, primary screening abnormality findings, colposcopy and histopathologic diagnosis attendance rates, and colposcopy abnormalities, rather than crude rates for comparison, to eliminate the effect of different age composition of the two groups. The results are summarized in Table 3.

**Table 3:** Comparison of the screening effectiveness of HPV-based strategy and cytology strategy.

Indicators	Cytology screening	HPV-based screening	*P* value
The age-standardized abnormality rate of primary screening (%)	2.8	3.4	*P*<0.001
The age-standardized attendance rate of colposcopy (%)	68.9	76.5	*P*<0.001
The age-standardized abnormality rate of colposcopy (%)	44.0	76.0	*P*<0.001
The age-standardized attendance rate of histopathological diagnosis (%)	78.0	94.1	*P*<0.001
The age-standardized detection rate of cervical precancerous lesions and cancer (per 100,000)	156.4	393.1	*P*<0.001

## Discussion

This cross-sectional population-based study analyzed the results of cervical cancer screening among women aged 35–64 years in Chengdu and showed that HPV-based screening strategy could effectively detect HPV-infected individuals and patients with cervical lesions, which is an effective way to achieve the tertiary prevention strategy for cervical cancer. Our study also demonstrated that HPV testing combined with cytology triage was more effective than cytology alone for large-scale population screening, with higher rates of abnormal primary screening, abnormal colposcopy findings, and cervical lesion detections.

### Screening findings about HPV infection and cervical lesions

HR-HPV infection is known to be the primary cause of cervical cancer [12]. Our study showed that the overall HR-HPV prevalence among women aged 35–64 years in Chengdu was 11.7%, with an HPV 16/18 infection rate of 1.4%. These findings were consistent with previous studies conducted by He et al. (12.6%, 1.7%) and Zhang et al. (13.2%, 1.3%) on the prevalence of HPV infection in Sichuan Province [13, 14]. We noted that the proportion of HPV 16/18 infections in women aged 35–64 years accounted for only 12.06% of the HR-HPV-positive population, which was attributed to the fact that HPV 52 and 58 were the predominant genotypes in the general population in Sichuan province, whereas HPV 16/18 had a higher proportion of infections in patients with cervical lesions, especially in high-grade lesions [15–19]. Unfortunately, due to the limitations of HPV testing kits, we were unable to confirm this possibility through HPV genotyping in this study.

In Chengdu, the age-specific rates of HR-HPV infection demonstrated an increasing trend with age, with a rapid increase in the prevalence of HPV among women older than 50 years old, with the highest prevalence in the 60–64 age group. These results align with the research of Liao et al. and Li et al., which both identified a higher incidence of HPV infection among women ≥ 50 years [18, 20]. The elevated prevalence of HPV infection in older women may be related to hormonal fluctuations and reduced immune function during menopause [21]. Interestingly, our findings on the age-specific prevalence of cervical lesions in the population showed that it did not increase with age, as did the prevalence of HPV infection, but rather showed an overall decreasing trend, with higher prevalence in younger women aged 35–44 years. This finding is consistent with other studies that have concluded that the risk of high-grade lesions decreases with age [22, 23]. This may be related to the fact that higher HPV infection rates in the older population are not all newly acquired infections, but mostly latent infections reactivated due to hormonal and immune disorders, which mostly cause low-level productive infection and did not cause evident cervical lesions [24, 25]. However, the younger trend of cervical lesions reminds us that regular screening in young women should not be neglected.

### Real-world effectiveness of different strategies and problems in screening

Cervical cancer screening in Chengdu has shown that HR-HPV testing combined with genotyping and cytology triage provides better screening effectiveness than cytology screening. First, our findings suggest that the age-standardized abnormality rates of primary screening results were significantly higher for HPV-based screening compared with cytology. This supports the conclusion that the HPV-based method is more sensitive than cytology as a primary screening method, could detect more people with suspicious cervical lesions and reduce the rate of underdiagnosis [26, 27]. Second, the proportion of abnormal colposcopy results following HPV-based primary screening was also much higher than that of cytology, indicating that HPV testing combined with genotyping and cytology triage for primary screening has higher accuracy, which may relatively improve the benefit of colposcopy in cases where there is a higher colposcopy referral rate for HPV-based testing [28, 29]. Finally, the HPV-based screening method demonstrated a significantly higher age-standardized detection rate of cervical lesions compared to cytology, especially its greater sensitivity to HSIL/CIN2-3, as reported in previous studies [30]. In addition, the age-standardized rates of colposcopy and histopathology attendance in the HPV-based screening strategy were higher than those for cytology, which may be related to the fact that HR-HPV-positive results caused more anxiety or fear of having cervical cancer among screeners, leading to increased referral rates [31]. A study of willingness to colposcopy referral also revealed that a positive HR-HPV result significantly increased attendance by three times [32].

There were some deficiencies of cervical cancer screening program in Chengdu. Firstly, the proportion of cytology triage in the non-16/18 HR-HPV positive population was inadequate. This could lead to lower rates of primary screening abnormalities and an increased number of missed cases with cervical lesions that would have been detected in the HPV-based screening strategy. Additionally, the attendance rates for colposcopy and pathology were far from the 90% required by the screening guidelines in Chengdu, contributing to an even lower detection rate of cervical lesions. Upon analyzing the timing of these participants’ screening, it was found that the lockdown policy implemented due to COVID-19 in 2021–2022 may have been a significant factor preventing women from receiving timely follow-up [33, 34]. The cervical cancer screening program in Chengdu should strengthen the management of participants with abnormal results in each procedure, following the screening workflow. It’s important to encourage them to undergo necessary follow-up examinations promptly, aiming to reduce the incidence and mortality cervical cancer.

### Advantages and limitations of this study

Our study is a real-world study, serving as an important supplement to randomized clinical trials, evaluating the effectiveness of different screening methods in a real community setting and large-scale population. In addition, this study’s large community-based population sample provides a more representative and thorough depiction of HPV infection and cervical lesions prevalence among women in Chengdu than many of the existing studies with smaller sample sizes or samples drawn from hospital patients [15, 17, 18, 35, 36]. There are also some limitations in this study. Firstly, our study only analyzed the results of the first round of screening, and participants with abnormal results requiring repeat screening 1–2 years later were not included. Secondly, the examinations and tests were conducted in several different hospitals, primary maternal and child health institutions, and third-party laboratory testing institutions, which may have led to some measurement and reporting bias, despite our efforts at quality control. Finally, due to the lack of other demographic characteristics of the screening population and the investigation of factors affecting screening willingness, the analysis only focused on the potential impact of age on screening results. Other factors such as economic status, education level, regional differences, and personal willingness have not been discussed, so the reasons for the different age composition of the two different screening strategies found in this study cannot be explained at the moment and need to be further investigated and analyzed in future studies.

## Conclusion

Our study demonstrates that the implementation of HPV testing combined with 16/18 genotyping and cytology triage significantly enhances the real-world effectiveness of cervical cancer screening, in comparison to the conventional cytology screening method. Our study provides valuable evidence and experience for advocating the adoption of HPV-based screening strategies in other cities in China or similar middle-income countries to accelerate the global elimination of cervical cancer.
